# Paget’s disease of bone with superimposed osseous metastasis from lung carcinoma: A case report

**DOI:** 10.1016/j.radcr.2026.07.084

**Published:** 2026-08-19

**Authors:** Andrew Gaetano, Arianna Smith, Claudia Rose Keating, Nicholas Wright, Elizabeth Sager, Emad Allam

**Affiliations:** Department of Radiology and Medical Imaging, Loyola University Medical Center and Loyola University Chicago, 2160 S 1st Avenue, Maywood, IL 60153, USA

**Keywords:** Paget’s disease of bone, Lung cancer, Bone lesion, Osteolytic lesion, Osseous metastasis, Pathologic fracture

## Abstract

Paget’s disease of bone is a common adult musculoskeletal disorder characterized by disorganized bone remodeling and abnormal bone turnover. Patients with Paget’s disease are predisposed to complications including bone pain, pathologic fracture, and malignant transformation. Superimposed metastatic disease within Paget-affected bone represents a diagnostic challenge due to overlapping imaging features. We present a case of a 63-year-old woman with known Paget’s disease of the right tibia who was subsequently diagnosed with metastatic non-small cell lung carcinoma (NSCLC). Imaging demonstrated a new lytic lesion in the right proximal tibia with cortical destruction and anterior soft tissue extension, consistent with metastatic disease on a background of Paget’s disease. Additional metastatic lesions were identified in the right superior pubic ramus/acetabulum, right posterior seventh rib, and right proximal humerus without associated Paget’s disease. The diagnosis of metastatic disease was further supported by biopsy confirmation of NSCLC origin from the acetabular lesion. Due to high fracture risk, the patient underwent prophylactic intramedullary nailing of the right tibia and later required intramedullary fixation of a pathologic right humeral fracture. This case highlights the importance of recognizing atypical imaging findings within Pagetic bone as timely diagnosis of superimposed metastatic disease can significantly impact management.

## Introduction

Paget’s disease of bone is a chronic metabolic bone disorder that most commonly affects older adults and is characterized by focal regions of accelerated bone turnover [1]. Excessive osteoclastic resorption followed by disorganized osteoblastic bone formation results in structurally abnormal bone that is enlarged, mechanically weak, and frequently hypervascular [2]. Patients may present with bone pain, deformity, or pathologic fracture; however, many individuals remain asymptomatic and may be diagnosed incidentally through elevated serum alkaline phosphatase levels or characteristic imaging findings [1,3].

Paget’s disease demonstrates a male predominance and increased prevalence with advancing age [1,4,5]. The disease is most frequently encountered in Europe, particularly in the United Kingdom and France [1]. Long-term complications include fracture, deformity, neurologic compression syndromes, and rarely, malignant transformation typically to osteosarcoma [[6], [7], [8]]. Metastatic involvement of bone affected by Paget’s disease is very uncommon and may pose a diagnostic challenge due to overlapping imaging features. In this case report, we describe a patient with lung carcinoma and an osseous lesion radiologically consistent with metastatic involvement of pre-existing Paget’s disease of the tibia, highlighting key imaging findings and diagnostic considerations.

## Case presentation

### Initial presentation and diagnosis

**Fig. 1:**
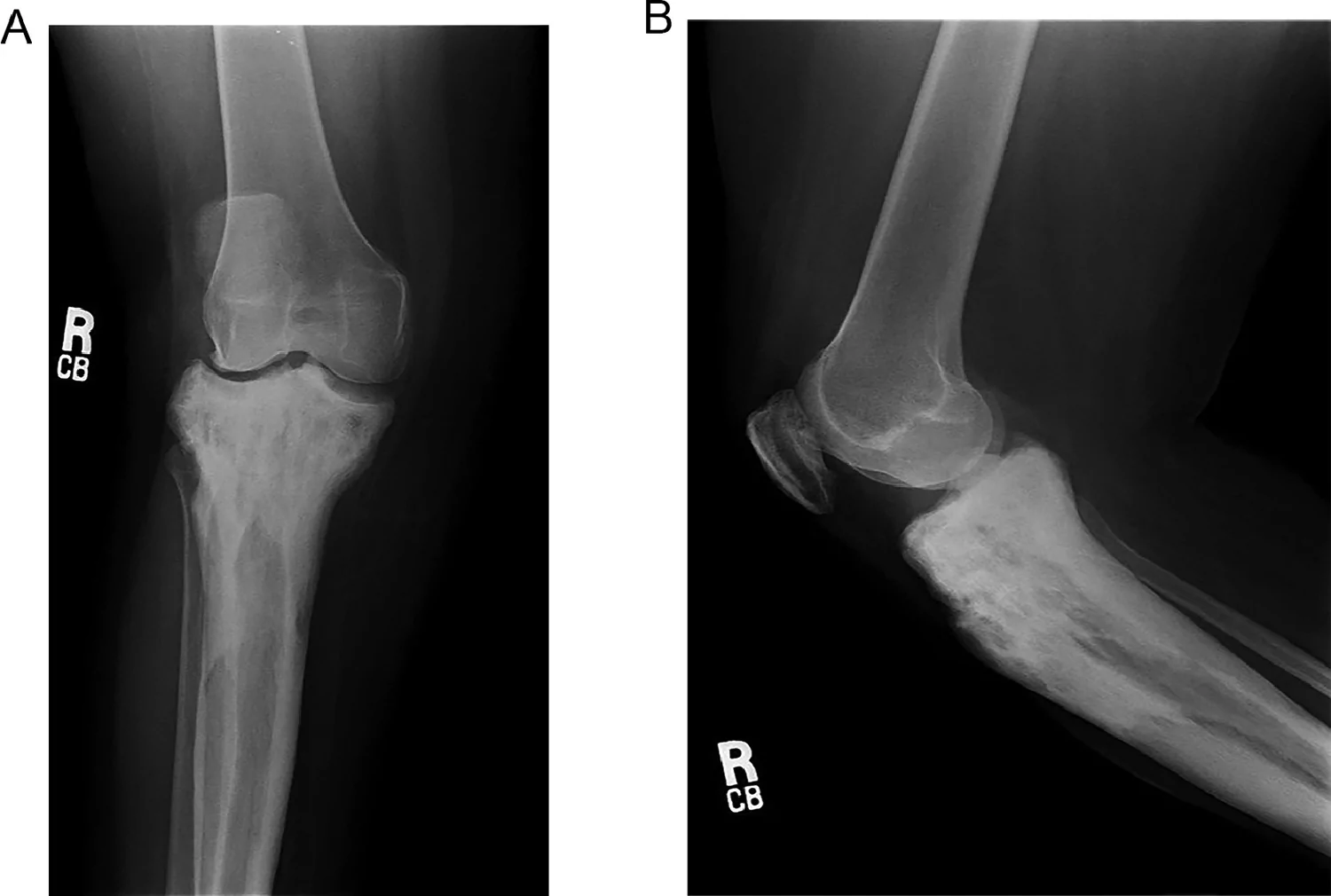
AP (A) and lateral (B) radiographs of the right knee demonstrate diffuse cortical thickening of the tibia as well as trabecular thickening/sclerosis of the proximal tibia including the tibial plateau, consistent with Paget’s disease of bone.

**Fig. 2:**
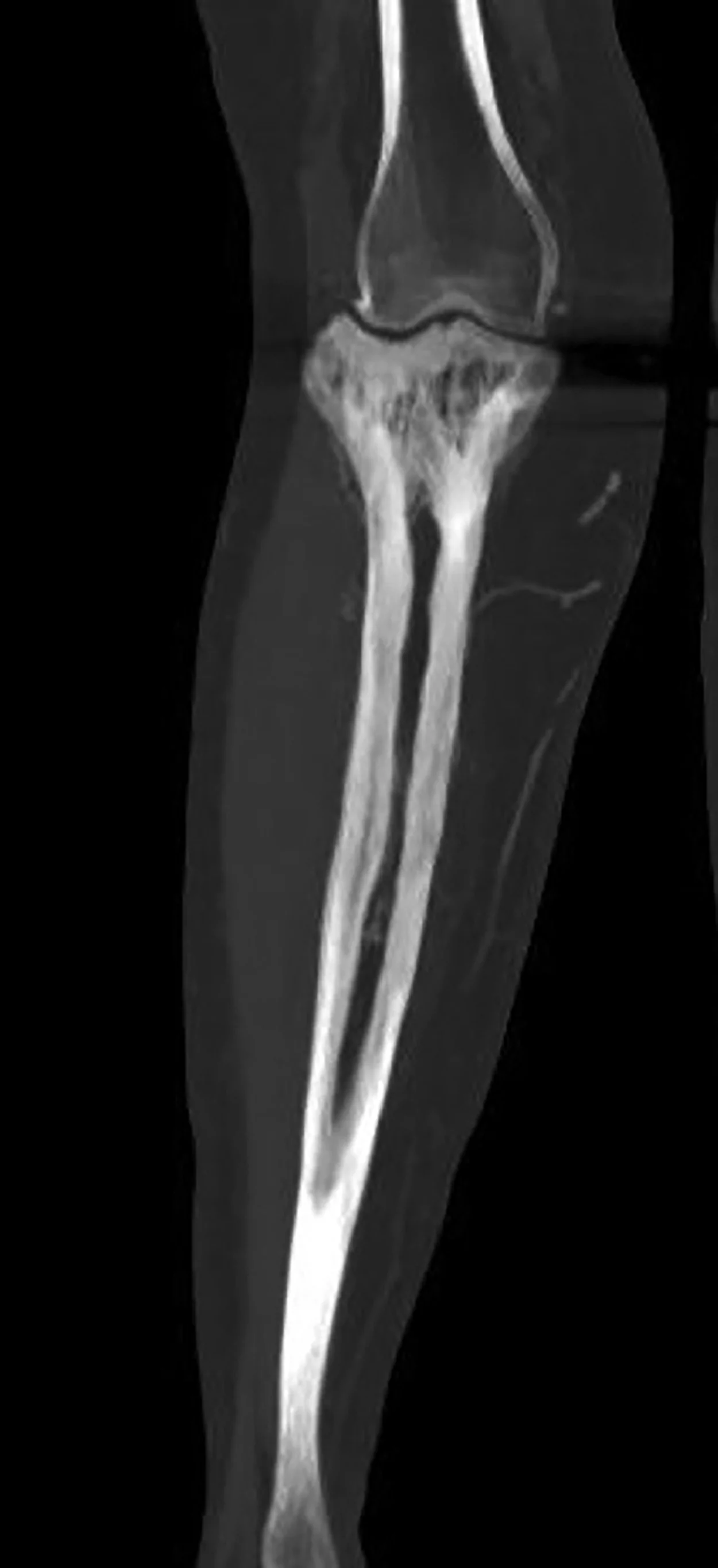
Coronal CT image confirms cortical and trabecular thickening of the right tibia, consistent with Paget’s disease of bone. No focal lucent lesion is seen.

**Fig. 3:**
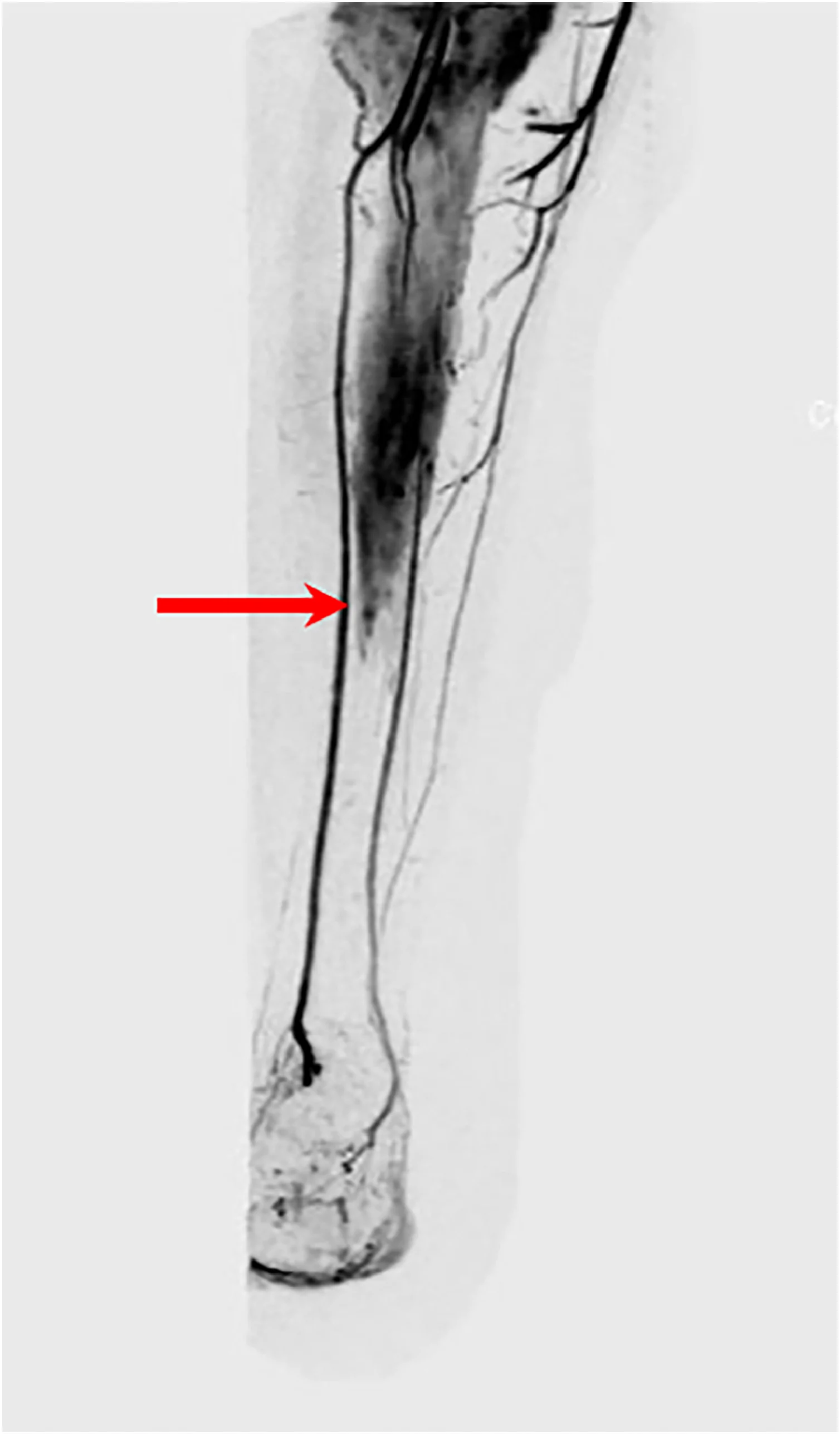
Reconstructed coronal MIP image from MR angiogram demonstrates marked hypervascularity of the right proximal to mid tibia corresponding to the region of Paget’s disease. There is a tapered appearance to the hypervascularity in the mid tibial diaphysis (arrow). This hypervascularity of Paget affected bone may facilitate hematogenous seeding by metastatic tumor cells.

**Fig. 4:**
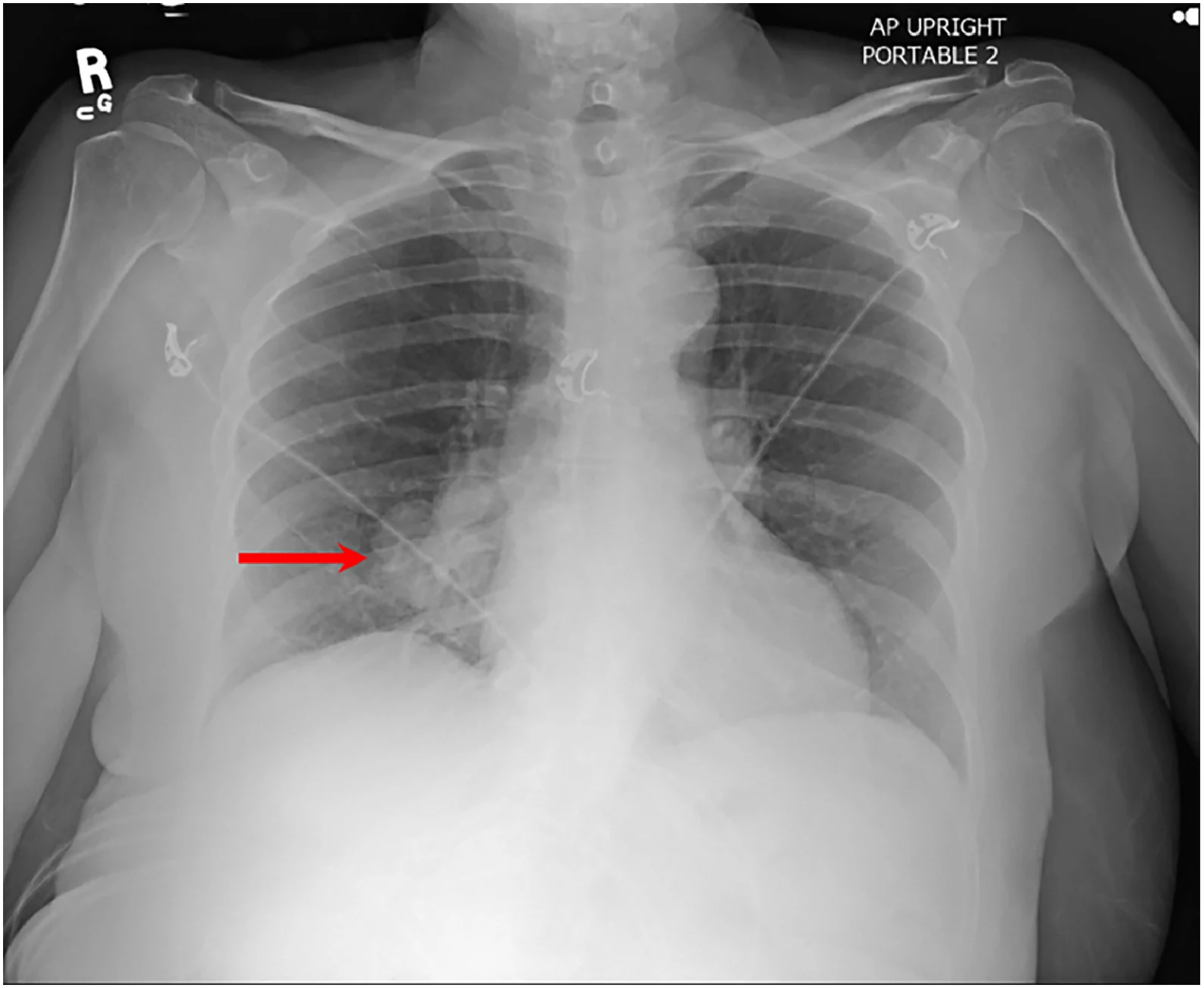
AP radiograph of the chest demonstrates a right infrahilar mass (arrow).

**Fig. 5:**
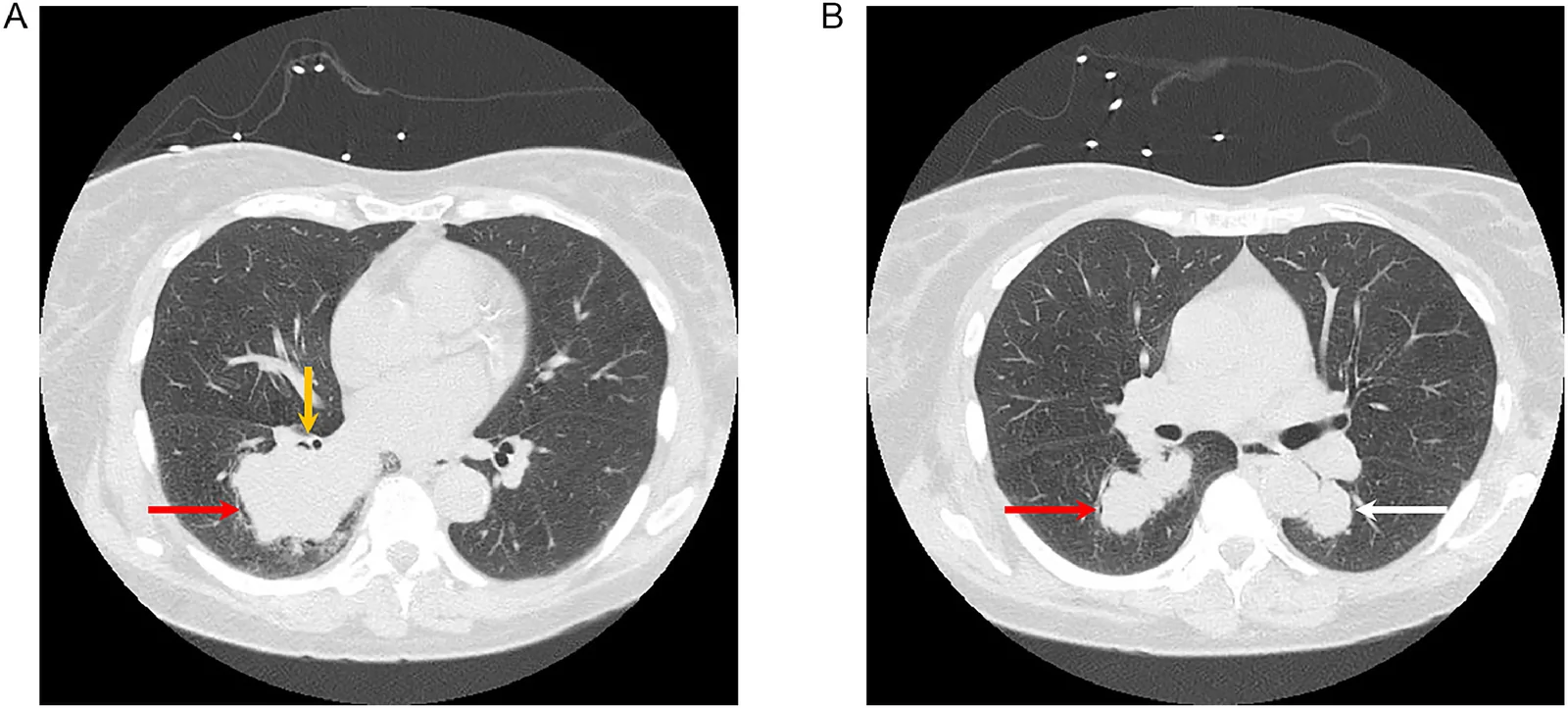
(A) Axial chest CT image demonstrates a large right lower lobe mass (red arrow), abutting/involving the bronchovascular bundle with partial effacement of a segmental bronchus (orange arrow). (B) Axial chest CT image (inferior to A) demonstrates a left lower lobe mass (white arrow) adjacent to the descending thoracic aorta, in addition to the right lower lobe mass (red arrow).

A 63-year-old woman with a history of tobacco use, chronic obstructive pulmonary disease, and known Paget’s disease of the right tibia (Fig. 1, Fig. 2, Fig. 3) presented with progressive shortness of breath. Chest radiograph and contrast-enhanced CT of the chest demonstrated bilateral lower lobe lung masses, greater on the right, raising concern for malignancy (Fig. 4, Fig. 5). Transbronchial biopsy subsequently confirmed non–small cell lung carcinoma.

### Development of osseous lesions

**Fig. 6:**
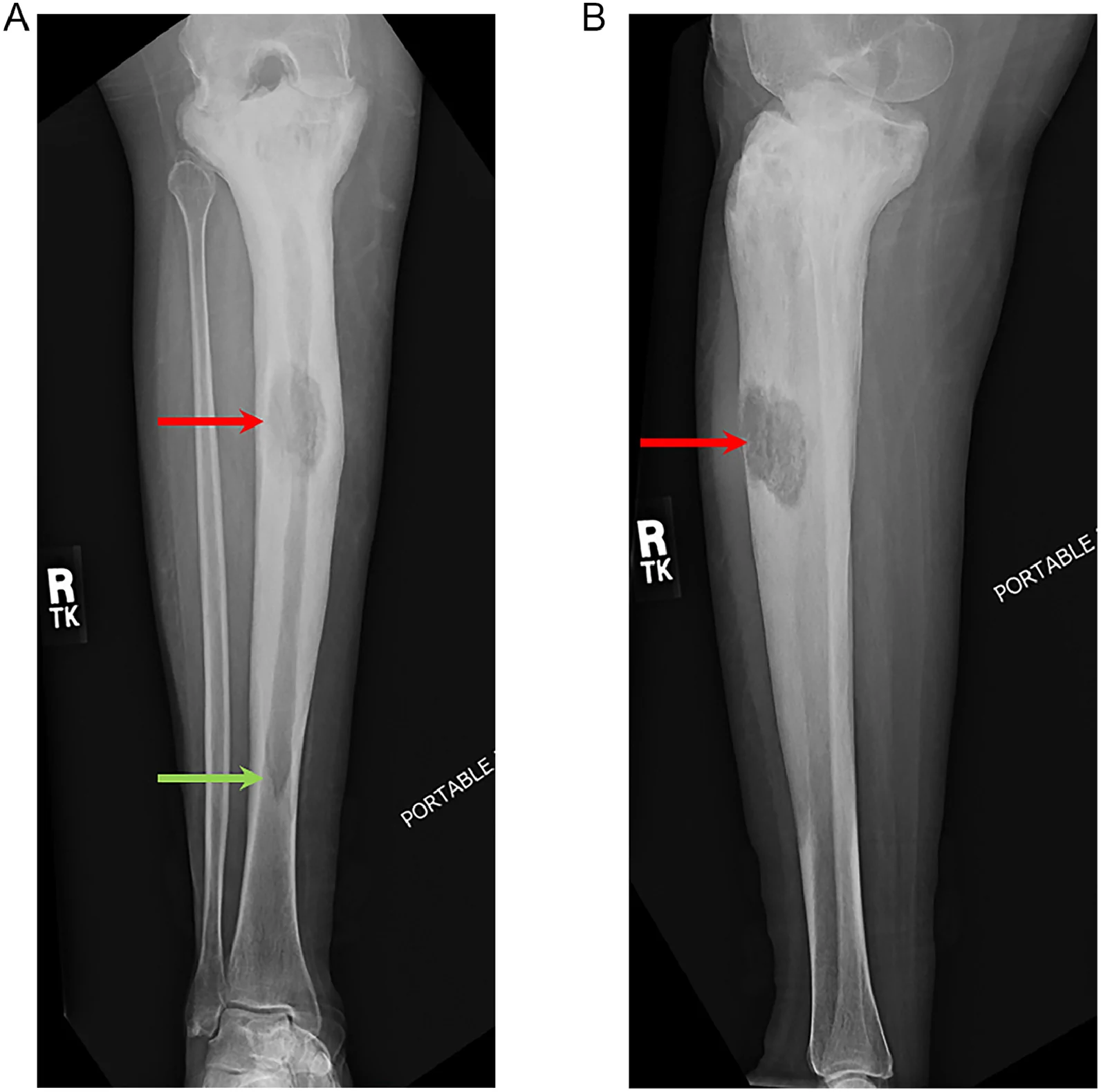
AP (A) and lateral (B) radiographs of the right tibia/fibula demonstrate extensive Paget’s disease of the tibia with demarcation indicated by the tapered lucency (“blade of grass” sign) at the distal tibial diaphysis (green arrow). Compared to Fig. 1, Fig. 2, there is a new lytic lesion in the proximal to mid tibial diaphysis with cortical thinning and discontinuity anteriorly (red arrow). No osteoid matrix is seen in the lesion.

**Fig. 7:**
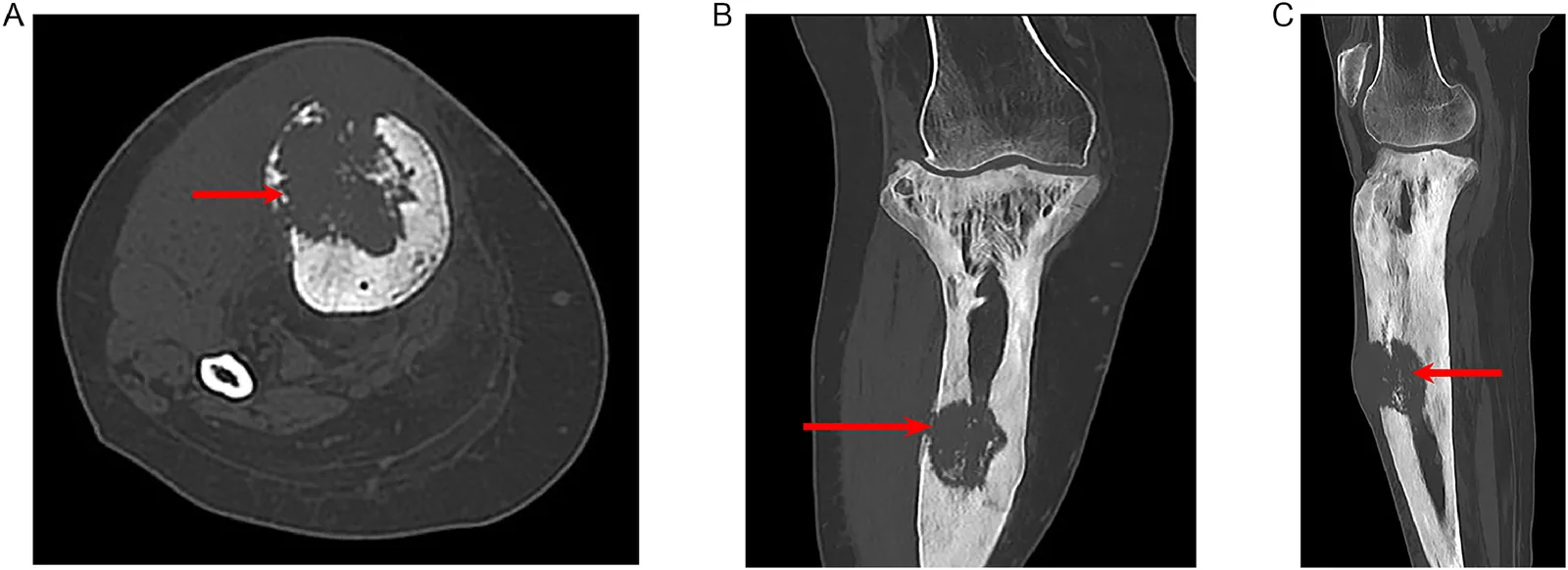
Axial (A), coronal (B), and sagittal (C) CT images demonstrate a lytic lesion in the proximal to mid tibial diaphysis with cortical destruction and soft tissue component anteriorly (arrow). There is diffuse cortical thickening of the tibia as well as trabecular thickening of the proximal tibia consistent with underlying Paget’s disease.

Approximately 4 months after the initial cancer diagnosis, the patient developed worsening pain in the right lower extremity. Radiographs of the right tibia demonstrated extensive Paget’s disease with a new lytic lesion in the proximal tibial diaphysis, associated with cortical thinning and focal cortical disruption (Fig. 6). CT was performed to further characterize this lesion, revealing a destructive lytic lesion with an associated anterior soft tissue component (Fig. 7). This lesion was suspicious for metastatic disease within Pagetic bone rather than a progression of Paget’s disease and represented the patient’s first site of osseous metastasis.

### Orthopedic management and disease progression

**Fig. 8:**
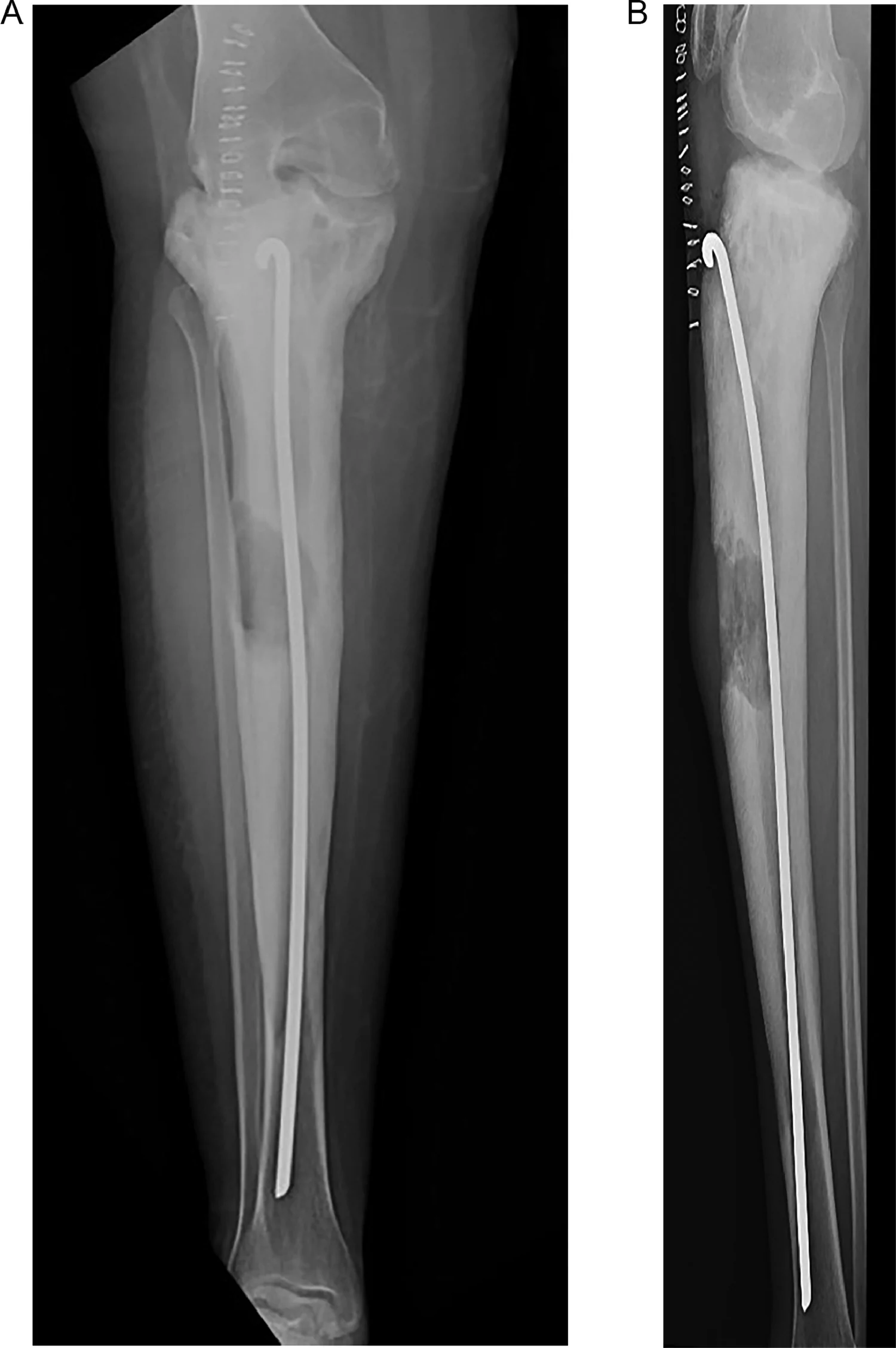
Postoperative AP (A) and lateral (B) radiographs of the right tibia/fibula demonstrate prophylactic intramedullary nailing of the right tibia performed due to the risk of pathologic fracture of the tibial lesion.

**Fig. 9:**
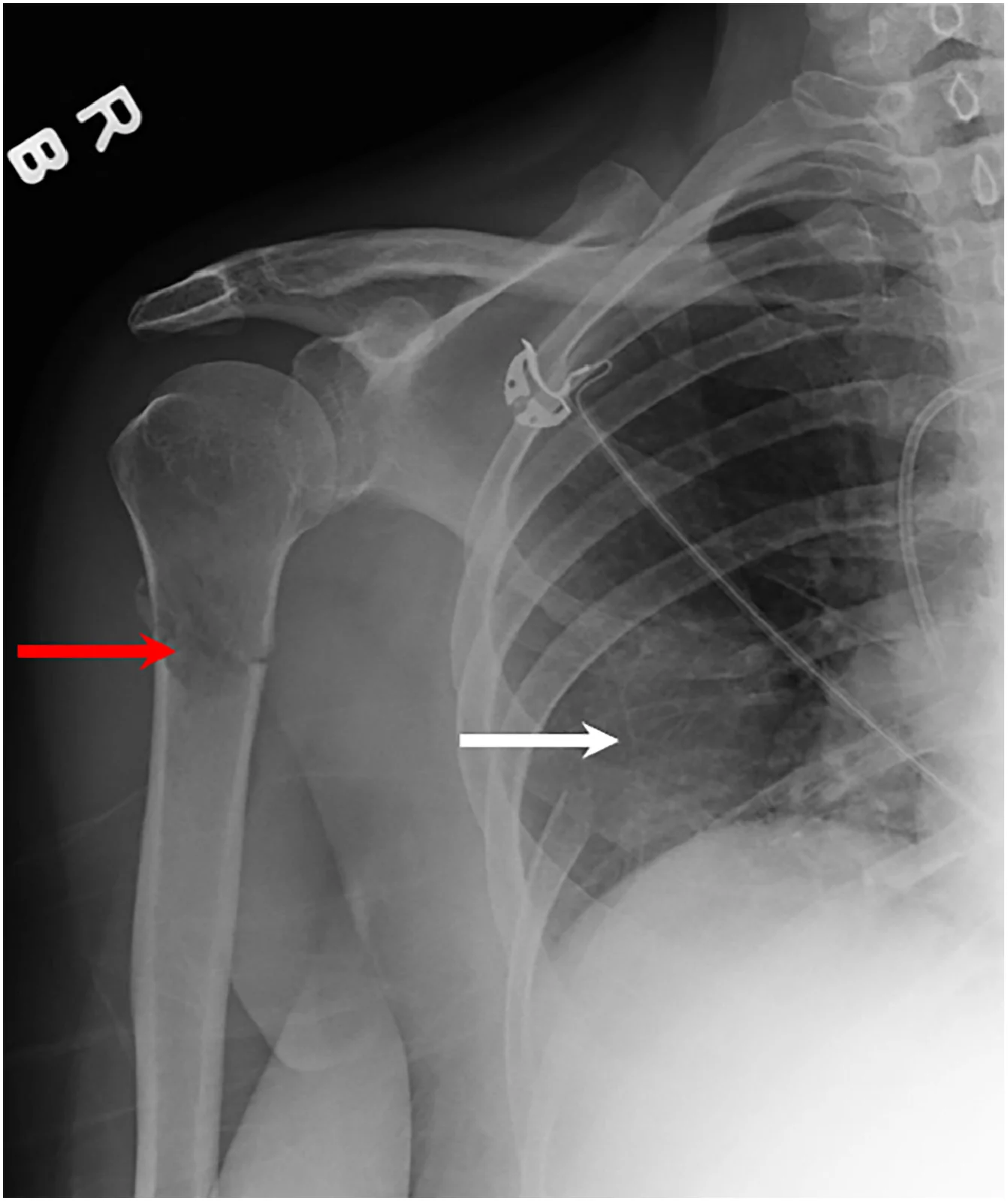
AP view of the right shoulder demonstrates a pathologic fracture of the right proximal humeral diaphysis with an underlying lytic lesion (red arrow). There is also destruction of the right posterior seventh rib (white arrow). The right infrahilar lung mass is again seen. A port catheter is partially imaged.

Given the high risk for impending pathologic fracture, the patient underwent prophylactic intramedullary nailing of the right tibia (Fig. 8). Subsequent imaging demonstrated additional lytic metastatic lesions involving the right superior pubic ramus/acetabulum, right posterior seventh rib, and right proximal humeral diaphysis. The patient later sustained a pathologic fracture of the right proximal humerus (Fig. 9), which was treated with intramedullary fixation. CT-guided biopsy of the right acetabular lesion was performed and was consistent with metastatic squamous cell carcinoma from a lung primary. Despite systemic chemotherapy and radiation therapy, the patient experienced progression of metastatic disease and was ultimately lost to follow-up.

## Discussion

Paget’s disease of bone is characterized by excessive, disorganized bone remodeling, resulting in structurally abnormal bone that is enlarged, mechanically weak, and hypervascular, thereby predisposing patients to fracture and other complications [1]. The disease most commonly affects the skull, pelvis, spine, femur, tibia, and proximal humerus. Although many patients are asymptomatic, clinical manifestations may include bone pain, deformity, hearing loss, or high-output cardiac failure related to increased vascularity [2,7].

Paget’s disease of bone classically progresses through osteolytic, mixed, and sclerotic phases, each associated with characteristic imaging findings [9]. The osteolytic phase demonstrates radiolucency due to increased osteoclastic activity. The mixed phase is marked by cortical thickening, osseous expansion, loss of corticomedullary differentiation, and accentuated trabeculae. The sclerotic phase is characterized by diffusely increased bone density. It is important to note that these phases may coexist within the same bone, complicating radiologic interpretation and potentially obscuring superimposed pathology [8].

Malignant complications of Paget’s disease most commonly involve sarcomatous degeneration, which occurs in fewer than 1% of cases [10]. Metastatic involvement of Pagetic bone from an extrinsic primary malignancy is exceedingly rare with reported primary sources including lung, breast, and prostate carcinoma [11,12]. A retrospective match-controlled study comparing patients with prostate cancer both with and without pre-existing Paget’s disease of bone found that patients with concomitant Paget’s disease and prostate cancer had a significantly increased time from diagnosis to osseous metastasis as well as increased overall survival [13]. This suggests that Paget’s disease could potentially exhibit a protective mechanism against osseous metastasis. However, this goes against the hypothesis that hypervascular Pagetic bone would be at an increased risk of hematological metastasis. These findings may be attributed to length-time or lead-time bias. In particular, patients with known Paget’s disease may undergo more frequent imaging surveillance for skeletal complaints, leading to earlier detection of both prostate cancer and subsequent bone metastases compared to patients without Paget’s disease. Further studies are necessary to better characterize the relationship between Paget’s disease and osseous metastasis as the literature remains limited in this area.

Distinguishing superimposed metastatic disease from Paget-related changes can be challenging, particularly when both entities demonstrate increased bone turnover and altered trabecular architecture. In the present case, several key radiologic features support the diagnosis of superimposed metastatic disease rather than an osteolytic component of Paget’s disease alone. Specifically, CT imaging of the right proximal tibia demonstrated a lytic lesion with focal cortical destruction and an associated anterior soft tissue component. Focal cortical destruction is a hallmark CT finding of osseous metastasis and has been found to be significantly associated with malignant origin on biopsy [14]. This finding particularly contrasts with the cortical thickening found in uncomplicated Paget’s disease, further supporting the metastatic nature of the tibial lesion [8]. Cortical destruction in combination with a soft tissue lesion on CT is a strong indicator of bony metastatic disease in patients with non-small cell lung cancer [15]. Malignant degeneration of Paget’s disease to osteosarcoma or fibrosarcoma was also considered less likely given known primary lung cancer and lack of osteoid matrix in the tibial lesion. T1-weighted MRI is the most clinically validated imaging sequence for distinguishing benign osteolysis in Paget affected bone from malignant transformation or superimposed metastasis. Signal intensities in Paget affected bone are most similar to those of fat, and this preservation of fat signal within bone on T1-weighted MRI has been found to effectively rule out neoplasm [16]. Conversely, replacement of normal fat signal on T1-weighted MRI by findings such as low T1 signal intensity with accompanying high T2 signal, gadolinium enhancement, and cortical destruction with an associated soft tissue mass is consistent with malignancy. Superimposed metastasis from a distant site may be indistinguishable from sarcomatous transformation of Paget disease on imaging alone without clinical context [17]. CT and MRI serve a critical role in guiding the clinical decision between conservative follow-up or biopsy/treatment of new or concerning bony changes identified in patients with Paget’s disease. While the international clinical guidelines do not recommend CT or MRI for diagnosis of Paget’s disease, they are recommended for assessment of disease complications including suspected neoplasm, as in the present case [1].

Despite the potential difficulty in differentiating between osteolytic Paget’s disease and metastatic bone lesions, differences on plain radiographs can help to distinguish these disease processes. For example, even in the osteolytic phase of Paget’s disease, cortical thickening can be observed, in contrast to metastatic lesions which often demonstrate cortical erosion [1,18,19]. Additionally, the osteolytic phase of Paget’s disease characteristically shows lesions with well-defined margins compared to the indistinct margins of metastatic bone lesions [1,19].

Adjunctive nuclear medicine studies may further aid in differentiation [1]. Paget’s disease typically demonstrates intense, symmetric radiotracer uptake on 99mTc-methylene diphosphonate bone scintigraphy corresponding to the involved bone, whereas metastatic disease more commonly presents as focal or asymmetric areas of increased uptake. When imaging findings remain equivocal, bone biopsy can be performed to establish the presence of superimposed metastatic disease.

## Conclusion

Metastatic involvement of bone affected by Paget’s disease is rare but clinically significant. Recognition of new or atypical lytic lesions within Paget-affected bone is critical, as timely diagnosis of superimposed metastatic disease can meaningfully influence management and prognosis. This case underscores the importance of careful imaging evaluation and multidisciplinary collaboration when assessing patients with Paget’s disease of the bone and known malignancy.

## Patient consent

Informed consent for this case was obtained from the patient’s next of kin.
